# Urban–Rural Disparities in the Transition to Parenthood During Times of Uncertainty: A Multilevel Perspective on Finland

**DOI:** 10.1007/s10680-024-09725-3

**Published:** 2024-12-13

**Authors:** Nicholas Campisi, Hill Kulu, Júlia Mikolai, Sebastian Klüsener, Mikko Myrskylä

**Affiliations:** 1 University of St Andrews, St Andrews, UK; 2 Max Planck Institute for Demographic Research, Rostock, Germany; 3 Federal Institute for Population Research (BiB), Wiesbaden, Germany; 4 Institute of Sociology and Social Psychology (ISS), University of Cologne, Cologne, Germany; 5 Vytautas Magnus University, Kaunas, Lithuania; 6 Helsinki Institute for Demography and Population Health, University of Helsinki, Helsinki, Finland; 7 Max Planck - University of Helsinki Center for Social Inequalities in Population Health, Rostock, Germany, Helsinki, Finland

**Keywords:** First birth, Uncertainty, Nordic, Register data, Spatial variation

## Abstract

Over the last 15 years, many European countries have experienced fertility declines. Existing research on this shift in fertility behavior points to economic aspects and increased levels of uncertainty as important drivers. However, in this debate little attention has been paid to how the relevance of individual- and contextual-level dimensions for understanding the new fertility patterns varies by level of urbanization. This is surprising given that urban and rural areas not only differ strongly in fertility timing and levels, but also in economic conditions. Our paper fills this important research gap by analyzing rich register data from Finland using multi-level event history models to study the transition to first birth among younger (under age 30) and older (ages 30 or older) women. We show that urban–rural differences in the transition to parenthood are particularly pronounced among younger women. In addition, the results indicate that economic circumstances and related uncertainties are more relevant for understanding first births probabilities for younger women than older women. Finally, among younger women, the relevance of economic circumstances and related uncertainties seems to be most relevant in the capital region of Helsinki and urban areas compared to semiurban and rural areas. Our findings underline that the urban–rural dimension should receive more attention in research on fertility in times of uncertainty.

## Introduction

In the aftermath of the 2008 economic crisis, many European countries experienced substantial fertility declines. Finland, the context of this study, experienced one of the largest fertility declines, not only among the Nordic countries but also in Europe. Explanations for this shift in fertility behavior point to economic aspects and increased levels of uncertainty as important drivers (Comolli et al., 2019; Goldstein et al., 2013; Matysiak et al., 2020; Miettinen & Jalovaara, 2020; Sobotka et al., 2011; Vignoli et al., 2020a).

While existing research has studied the relevance of both individual- (e.g., employment, income, or education) and contextual-level (e.g., municipality unemployment rate, total fertility rate, or level of urbanization) factors for understanding recent shifts in fertility behavior, little attention has been paid to how the roles of these factors vary by level of urbanization. This is surprising as existing research provides evidence for strong systematic disparities in fertility levels and timing between urban and rural areas (e.g., Campisi et al., 2020, 2022; Kulu, 2013; Kulu et al., 2007; Nisén et al., 2020; Vitali & Billari, 2017). The transition to parenthood occurs on average much earlier in rural compared to urban areas (Kulu et al., 2007). This is of relevance as one option to cope with uncertainty is to postpone births to later ages.

Economic conditions also tend to differ strongly between urban and rural areas. Urban areas usually have more diverse labor markets, which are more resilient to economic shocks compared to rural areas (Blank, 2005). Economic factors can also disproportionately impact fertility at different ages (Jalovaara & Miettinen, 2013; Miettinen & Jalovaara, 2020) which can have implications for urban–rural disparities. For example, unemployment among women at younger childbearing ages may be more detrimental to their fertility aspirations than for older women who may have more resources accumulated over time. This might particularly be the case in urban areas in which living costs are higher compared to rural areas, which might discourage fertility (Kulu & Washbrook, 2014; Kulu et al., 2007; Livi-Bacci & Breschi, 1990), especially after economic shocks (Kulu, 2013).

Our paper contributes to closing existing knowledge gaps related to the role of urbanization in the uncertainty–fertility relationship. To do so, our primary research question is how the transition to first birth varies between urban and rural areas during times of economic uncertainty. Transitions to first births are particularly well suited in this regard as it has been shown that young adults are particularly affected by increased economic uncertainty (Goldstein et al., 2013; Vignoli et al., 2012). To answer this question, we analyze the transition to first birth in Finland between 2012 and 2018, using rich, highly detailed register data. In light of the above-mentioned findings that increased uncertainty might be more relevant for fertility decisions of young adults, we put a special emphasis on differences between women of younger and women of older childbearing ages.

This paper aims to further understand variation of fertility in Finland across levels of urbanization and what, if any, level of urbanization plays in previously established economic uncertainty, namely unemployment, theories. Our study provides three key contributions to the literature. First, in the theoretical section, we link literature strands dealing with (a) uncertainty and fertility and (b) urban–rural disparities in fertility to motivate why the urban–rural dimension is highly relevant for research on fertility in times of uncertainty. Second, we estimate multi-level models allowing us to explore how factors on both the individual-, such as economic activity or household income, and municipal contextual-level, such as level of urbanization or unemployment ratio, are related to individual-level fertility outcomes. We also estimate these models separately for younger (under age 30) and older women (ages 30 or older) to explore whether economic circumstances and related uncertainties are more relevant for understanding variation in first birth probabilities among younger women compared to older women. Third, we investigate whether the association between economic activity and the probability of a first birth varies across level of urbanization among younger and older women by including interaction effects between women’s economic activity and their municipality’s level of urbanization.

## Transition to a First Birth During Times of Uncertainty

### The Individual-Level Perspective

Unemployment (Busetta et al., 2019; Miettinen & Jalovaara, 2020; Vignoli et al., 2020a; Vikat, 2004), insecure employment (Adserà, 2011; Alderotti et al., 2021; Schmitt, 2012) or otherwise low confidence in the economy (Comolli, 2017) can all deter entry into parenthood as individuals seek to protect themselves against further economic hardship, especially if it is prolonged (Busetta et al., 2019). After the 2008 economic crisis, theories of uncertainty gained new relevance as unemployment levels increased in many countries and many individuals were forced to reconsider their plans for the future, including their childbearing intentions (Comolli et al., 2019; Sobotka et al., 2011).

Economic instability might compete with having children in multiple ways (Friedman et al., 1994). First, children can be seen as a barrier to finding employment for unemployed individuals (Adserà, 2011). Having a child without secure employment may limit individuals’ means of increasing employability, such as gaining additional skills or qualifications (Adserà, 2004). Second, reentering the labor force full-time after childbirth may be difficult and childrearing may have negative consequences for women’s career and earnings potential (Rønsen & Sundström, 2004). Third, persistent unemployment competes financially with having children by reducing long-term financial savings due to unearned or lost income (Adserà, 2011; Brand, 2015), thus reducing financial resources available for children. For women already unsure about parenthood, unemployment may only be one aspect of the decision to remain childless. Personal motivations, such as lifestyle changes or having other priorities in life (Miettinen, 2010; Miettinen & Paajanen, 2005), for postponing childbirth may compound with unemployment.

However, supplemented income may reduce the negative relationship between economic uncertainty and fertility (Alderotti et al., 2021). Social welfare or income from partners reduce the economic barriers to fertility (Rønsen, 2004), especially if the male partner is employed (Busetta et al., 2019; Santarelli, 2011; Vignoli et al., 2012). At the same time, some women with fewer educational or career alternatives to fertility may not be dissuaded from childbearing by economic uncertainty and may enter parenthood at younger ages (Kreyenfeld & Andersson, 2014; Miettinen & Jalovaara, 2020; Rondinelli et al., 2010). Children, instead of education or career development, may serve as a source of structure or life satisfaction for women with low levels of education (Kreyenfeld, 2010; Miettinen & Jalovaara, 2020).

Alternative pathways to adulthood other than parenthood (Liefbroer & Toulemon, 2010) may also contribute to ambivalence toward parenthood and encourage fertility postponement at younger ages (Bernhardt & Goldscheider, 2014). Younger women have more flexibility, in part biologically, than older women to postpone childbearing until they want to become mothers. A typical strategy among young women to address economic uncertainty is to invest in education, which often results in the postponement of childbearing (Kreyenfeld, 2005). This disproportionate impact of economic uncertainty on fertility by age may be one reason why supplemented incomes are more related to entry into parenthood for younger women than for older women (Jalovaara & Miettinen, 2013). The discussed mechanisms contribute to the situation that fertility at younger ages is increasingly being associated with economic insecurity and low socio-economic status (Miettinen & Jalovaara, 2020; Sigle & Kravdal, 2021).

However, fertility behavior often relies not only on immediate or measurable events such as unemployment, but also on broader schemas or expectations which may not always be conscious (Morgan & Bachrach, 2011). Vignoli et al. (2020b) suggest that increasing childlessness in many European countries since 2008 may be related to individuals’ broader expectations as they build narratives about the future, which may extend long past economic crisis. Stagnant incomes (OECD, 2020) and stagnant economic growth since 2008 (World Bank, 2010) in some countries may contribute to decreased confidence in the broader economic situation and, consequently, contribute to lower fertility (Comolli, 2017; Fokkema et al., 2008). In this sense, even employed individuals living in regions with elevated levels of unemployment may be discouraged from childbearing (Kravdal, 2002). Subjective interpretations of objective measures, such as employment statistics or other measures of economic health, further add to the complexity in the link between economic uncertainty and fertility.

### The Urban–Rural Dimension in Times of Uncertainty

An urban–rural pattern of fertility, which can be generalized as lower fertility in urban areas and higher fertility in rural areas, is longstanding in Europe. During the last 50 years, differences in total fertility between more urbanized and more rural regions have become stronger (Kulu et al., 2007; Vitali & Billari, 2017) as fertility declined faster in urban than in rural regions (Kulu et al., 2007). Major cities continue to display lower fertility than the neighboring areas in recent years (Campisi et al., 2020).

The conditions of fertility variation across space are multidimensional, and how these dimensions are related to fertility vary by region and by level or urbanization (Campisi et al., 2020, 2022). This is also true when we look at spatial variation in uncertainty. Just as countries were unevenly impacted by the economic crisis (Goldstein et al., 2013), so were subnational regions (Matysiak et al., 2020; Puig-Barrachina et al., 2019; Sabater & Graham, 2018). Regional populations across Europe responded disproportionately to economic uncertainty in terms of fertility due to differences in their economic structure and resilience. Urban areas tend to be more resilient to shocks such as economic downturns as their economies tend to be more diverse (Blank, 2005). Thus, they may provide unemployed individuals with new employment opportunities quickly through flexible economies. In rural areas, on the other hand, economies tend to be less diverse (Blank, 2005). As a result, if an important economic sector is hit, this usually has tremendous implications for the local economy.

On the other hand, urban areas are usually characterized by higher living costs compared to rural areas (Kulu et al., 2007; Livi-Bacci & Breschi, 1990). Losing economic stability in a region with high costs of living will have a greater impact on daily life and financial decisions than in regions where the costs of living are lower (Kulu, 2013). The financial costs of housing, food, entertainment, or education may directly compete with the financial costs of children (Becker, 1991), especially if these costs are high. Thus, lost income through unemployment in a city might cause more economic hardship to an individual or household compared to lost income in a rural area, with potential implications for fertility decisions. At the same time, perceived uncertainty among employed persons might be higher in urban compared to rural areas due to the higher living costs. Overall, the higher living costs tend to make it more difficult for individuals, particularly for young people, to establish themselves in bigger cities (Kulu & Washbrook, 2014). Recent research shows that an uncertain life situation was a greater reason to postpone or not have more children for individuals living in the metropolitan area of Helsinki than other regions of Finland (Savelieva et al., 2022).

Migration is also relevant when looking at the link between economic conditions and fertility. Younger individuals tend to be more highly mobile than older individuals due to education, starting new employment, or joining a partner. High spatial mobility at younger ages may encourage women to wait to have a child until after a move (Andersson, 2004; Kulu & Vikat, 2007; Milewski, 2007) or to adapt to the fertility patterns of the receiving region (Lundström & Andersson, 2012). During times of crisis, individuals may migrate from regions, or even countries, with high unemployment levels to regions (or countries) with low unemployment levels in search of work. This migration has a negative impact on fertility in sending regions, further contributing to deflating the fertility levels of those areas (Sabater & Graham, 2018). In this sense, fertility decline in receiving regions will be mitigated by migrant fertility, while fertility in sending regions will further decline as younger individuals leave and have births elsewhere. On the other hand, selective migration may contribute to decrease fertility levels in receiving areas if it is driven by decisions to invest in education during times of uncertainty. These receiving areas are usually urban areas as educational institutions are mostly concentrated in cities. As fertility is usually postponed until after finishing education (e.g., Kulu & Washbrook, 2014), such coping strategies would contribute to depressing fertility in receiving urban areas.

### The Finnish Context

Finland’s economy fared relatively well during the global economic crisis and fertility levels remained relatively stable directly after 2008. However, fertility in Finland declined substantially after 2010 and became the lowest among the Nordic countries (Hellstrand et al., 2021). Childlessness in Finland has increased in recent cohorts (Jalovaara et al., 2019; Rotkirch & Miettinen, 2017) and is among the highest in Europe (Miettinen et al., 2015). Most (76 percent) of the change in fertility levels between 2010 and 2018 was explained by reductions in first births, whereas a reduction in second and third births only explained 13 and 8 percent of the total fertility decline, respectively (Hellstrand et al., 2020, 2021).

In Finland, the connection between unemployment and fertility was complex even before 2008. While women over age 30 who were unemployed displayed lower risks of a first birth than employed women, this trend was the opposite among women aged 20 to 30 years old (Vikat, 2004). The relatively strong social support in Finland may help mitigate the relationship between unemployment and fertility. For example, supplemented incomes from social benefits have been positively tied to fertility in recent years (Miettinen & Jalovaara, 2020) and historically (Berninger, 2013; Vikat, 2004). Although the reasons for using benefits may vary, individuals who utilize social benefits can display accelerated fertility (Aassve & Lappegård, 2009). However, the positive effect of supplemented income may not be long-term as unemployment benefits are not large (Miettinen & Jalovaara, 2020).

Women in Finland with lower levels of education appear to form a select group with fewer barriers to fertility. Unemployed women aged 18 to 24 with basic levels of education displayed a much higher risk of a first birth than their employed counterparts (Miettinen & Jalovaara, 2020). At other educational levels, the difference in the risk of a first birth between employed and unemployed women was small to none. Miettinen and Jalovaara (2020) attributed the increased first birth probabilities among the low educated to selectivity. Young women with low levels of education are a select group with lower opportunity costs to childbearing. At young ages, women are leaving school, enrolling in university, or just beginning their employment careers. For young women not enrolling in further education or employment, it may thus be easier to have a child during a period of short-term unemployment without upsetting their life plans, especially if lost incomes are supplemented. In this way, the strong Finnish social support system may reduce education- or employment-fertility tradeoffs among young women by preventing unemployment or other economic hardship from disrupting family formation patterns (Miettinen & Jalovaara, 2020).

Clearly, level of urbanization plays a role in Finnish fertility trends. Spatial variation of fertility between Finnish regions is among the highest of low-fertility countries in Europe (Campisi et al., 2020). Generally, fertility differences between regions exhibit an urban–rural gradient, in which fertility in urban regions is lower than fertility in rural regions (Campisi et al., 2020; Kulu et al., 2007, 2009). These fertility level differences between bigger regions tend to be larger than differences by socioeconomic groups within regions. For example, the difference in cohort fertility between the urban Helsinki-Uusimaa region and the rural North and East Finland region is almost twice as large as the variation in cohort fertility between educational groups within Helsinki-Uusimaa (Nisén et al., 2020).

Finland is a unique context of fertility and economic uncertainty given the country’s strong social welfare support and rapid fertility decline. In the Finnish context, we expect economic circumstances and related uncertainty to be negatively linked to fertility. We assume this to be particularly true for younger women as they are less established in life and have more options to postpone fertility to later ages compared to older women. However, the strong welfare state support might moderate this relationship. In light of these considerations, we expect the way in which economic circumstances and related uncertainty are linked to fertility to vary by level of urbanization in such a way that unemployment is more related to fertility in rural areas than urban areas, as it may be less financially draining but more prolonged. We also expect to find lower rates of first births in urban regions than in rural regions, in line with an urban–rural gradient of fertility levels. However, it is unclear from our theoretical considerations whether economic uncertainty is more relevant in rural or urban areas, as we identified potentially counteracting mechanisms.

## Data

We use yearly individual-level data from the Finnish population register for the period 2012 to 2018. Data on individuals are available for the years individuals lived in Finland and are provided by Statistics Finland. We use data for women aged 18 to 49 in any given year between 2012 and 2018. Data from the end of the previous year are used to calculate the probability of a birth between the years 2012 and 2018 unless otherwise noted. For example, 2012 data reflect information in the last week of 2011. We restrict the sample to omit women who had a first birth before 2012 and those which had a first birth outside of Finland to arrive at a final sample of 691,687 women. Table 1 shows the distribution of the sample by covariate categories. Some variables change over time, so Table 1 only reflects data for the first year a woman is in the sample. We note that the majority of our sample is aged 18 to 29. This is related to the fact that many women over age 30 were removed from the sample because they already experienced a first birth. Further information on data used in this paper, including more details on the measurements and calculations, is available in Appendix A.

**Table 1:** Sample distribution of first births and female population aged 18–49, 2012–2018. *Source*: Population register of Finland

Variable		First births	Women (percent)		Person years
Total		153,239	691,687 (100.0)		3,414,817
Economic activity	Employed	119,970	387,571 (56.0)		2,251,528
	Unemployed	13,293	37,507 (5.4)		268,085
	Student	13,432	215,706 (31.2)		611,922
	Not in labor force	6,544	50,903 (7.4)		283,282
Household income (Euros)	Less than 20,000	13,964	322,489 (46.6)		897,053
	20,000 – 38,000	39,078	175,658 (25.4)		1,091,031
	38,000 – 50,000	40,713	95,426 (13.8)		673,987
	50,000 – 64,000	36,318	6,1693 (8.9)		472,779
	64,000 or more	23,166	3,6421 (5.3)		279,967
Education	Low	21,599	296,180 (42.8)		632,172
	Medium	61,174	242,105 (35.0)		1,693,172
	High	70,466	153,402 (22.2)		1,089,473
Country of birth	Native	135,582	617,370 (89.3)		3,117,330
	Foreign born	17,657	74,317 (10.7)		297,487
Moved in year	Did not move	135,189	586,745 (84.8)		2,956,845
	Moved	18,050	104,942 (15.2)		457,972
Age	18–19	6,606	248,105 (35.9)		429,770
	20–24	34,522	151,269 (21.9)		1,022,059
	25–29	54,253	115,825 (16.8)		769,849
	30–34	40,593	66,828 (9.7)		462,606
	35–39	14,506	39,208 (5.7)		277,699
	40–44	2,584	34,615 (5.0)		219,425
	45–49	175	35,837 (5.2)		233,409
Municipality-level urbanization	Helsinki	25,137	117,749 (17.0)		671,246
	Urban	95,028	422,321 (61.1)		2,112,961
	Semiurban	18,496	81,666 (11.8)		345,827
	Rural	14,578	69,951 (10.1)		284,783
		Mean		Standard deviation	
Municipality unemployment ratio		0.12		0.04	
Municipality total fertility rate		1.59		0.33	
Municipality population density (population per km^2^)		0.78		1.09	

Distribution of women refers to their first year in the sample

### Variables of Analysis

Birth histories are constructed by linking children to their parents in the population register. Birth order is established using data on children’s birth month and year. There were 153,239 first births in Finland during the study period.

We measure individual-level socioeconomic conditions using information on economic activity, household income, and educational attainment. As we cannot fully capture all aspects of economic uncertainty, we focus on the status of being unemployed we interpret as a proxy for uncertainty, in line with previous studies examining the role of uncertainty for fertility in Finland (Miettinen & Jalovaara, 2020). As a contextual proxy of uncertainty, we use the proportion unemployed at the municipality level. As we expect unemployment to vary by level of urbanization, this is another important contextual variable. We also control for several basic demographic characteristics such as country of birth (native or foreign born), whether an individual moved in the year prior to first childbirth, age, as well as municipality-level total fertility rate, municipality-level population density, and year of observation.

Economic activity is measured as the main type of economic activity. It is categorized into employed, unemployed (unemployed or on unemployment benefit), student (student, pupil), or not in the labor force (pensioner, conscripted or performing community service, or others outside the labor force). Statistics Finland classifies individuals who do not have a job but are actively looking for one as unemployed. Those who do not have a job and are not actively looking for one are considered outside of the labor force.

Household income reflects the net income of a woman and her partner, if she has a partner who is co-residing with her. Net income is calculated as the gross income (sum of earned income, entrepreneurial income, property income, national pensions, other social security benefits or allowances, and other current transfers all received by the end of the previous year) minus taxes and other government charges. Household income is calculated as the sum of the net income of an individual and the net income of the partner. Partners are linked using information on co-residence established by Statistics Finland for couples who have lived together for at least 90 days. The income of unrelated individuals living in the same household is excluded from household income calculations. Net income, rather than gross income, is used to better reflect the income available to individuals. It also better accounts for differences in taxation or levies between individuals, between municipalities, and between different levels of gross income. It is measured in quintiles: less than 20,000 Euros; 20,000–38,000 Euros; 38,000–50,000 Euros; 50,000–64,000 Euros; and 64,000 Euros or more.

Educational attainment reflects the woman’s highest educational attainment. Education is measured as low (less than upper secondary), medium (upper secondary level or post-secondary, non-tertiary), or high (short-cycle tertiary, bachelor’s degree or equivalent and higher).

The municipality’s level of urbanization is measured for an individual’s municipality of residence. It is used as a proxy for aggregate-level factors related to first births in Finland that systematically vary by level of urbanization. We use a variant of the classification developed by Statistics Finland and differentiate between four types of regions: Helsinki, urban, semiurban, and rural areas. Helsinki is considered separately from urban municipalities due to its unique nature as the most densely populated city and the capital city. Levels of urbanization for each municipality are constant during the period, although an individual’s municipality of residence may change. In total, we use 310 municipalities in Finland.

The proportion unemployed at the municipality-level reflects large-scale economic conditions that may impact individuals’ fertility decisions. The proportion unemployed in each municipality is calculated as the proportion of unemployed individuals aged 18 to 49 in the municipality to the total municipality population aged 18 to 49.

The aggregate-level controls at the municipality level are population density and total fertility rate. Population density, calculated as inhabitants per km^2^, is included in the analysis to control for population density variation within areas with the same level of urbanization. We use the natural log of population density to reduce the impact of outliers. The total fertility rate at the municipality level reflects aggregate-level trends in fertility that may impact individuals’ fertility decisions.

## Methods

### Age-Specific Fertility of First Births

First, we examine age-specific fertility of first births to study variation in first birth rates by level of urbanization and for each economic activity status. First birth rates are calculated for the age range 18 to 49 by dividing the number of live births at each age by the total number of childless women at that age living in Finland between 2012 and 2018.

### Estimating the Hazard of a First Birth

Second, the probability of a first birth to women living in Finland between 2012 and 2018 is estimated using discrete-time event history models. We focus on women who were at risk of experiencing a first birth during the observation period, i.e., women who did not have a first birth before 2012 and were aged 18 to 49 in any given year during the study period.

We estimate discrete-time models using a person-year dataset with one observation for each individual and each year they are observed. Person-year observations are censored and removed for years when an individual does not live in Finland, after a first birth occurs, when an individual is older than 49, or when the observation period ends. Individuals who leave the sample but return in a later year (i.e., emigrate from Finland but return to Finland) are included only for the years in which they live in Finland. Individuals who leave Finland but return during the same year are included in the sample if they were present at the time of data collection and thus have data for that year. Further information on discrete-time event history analysis used in this paper can be found in Appendix A. As individuals are nested in municipalities, we estimate multi-level discrete-time event history models.

First, we estimate multilevel discrete-time event history models for women of all ages 18 to 49 to understand how individual- as well as aggregate-level factors are related to first birth transitions. Then, we estimate separate models for younger (aged 18–29) and older (ages 30–49) women to investigate whether and how model outcomes vary by age. In this comparison, we particularly focus on the outcomes of variables that are related to spatial variation and uncertainty considerations.

### First-Birth Hazard by Economic Activity and Level of Urbanization

As a third and final analytical step, we estimate the probability of a first birth by economic activity for each level of urbanization among younger and older women. To do so, we again calculate separate models by age group but interact the individual-level economic activity variable and the municipality-level variable level of urbanization. The aim is to explore whether the association between economic activity status and the probability of a first birth varies by level of urbanization. For example, is the probability of a first birth to unemployed women higher or lower in urban municipalities than in rural municipalities? This model controls for all the individual- and municipality-level characteristics included in the previous models. We omit students from these results, but not the model, because they are often clustered at universities in urban centers and their fertility may be impacted by their municipality of birth or where their family lives, rather than the municipality in which they reside as students.

## Results

### First Birth Rates by Urbanization and Economic Activity

**Fig. 1:**
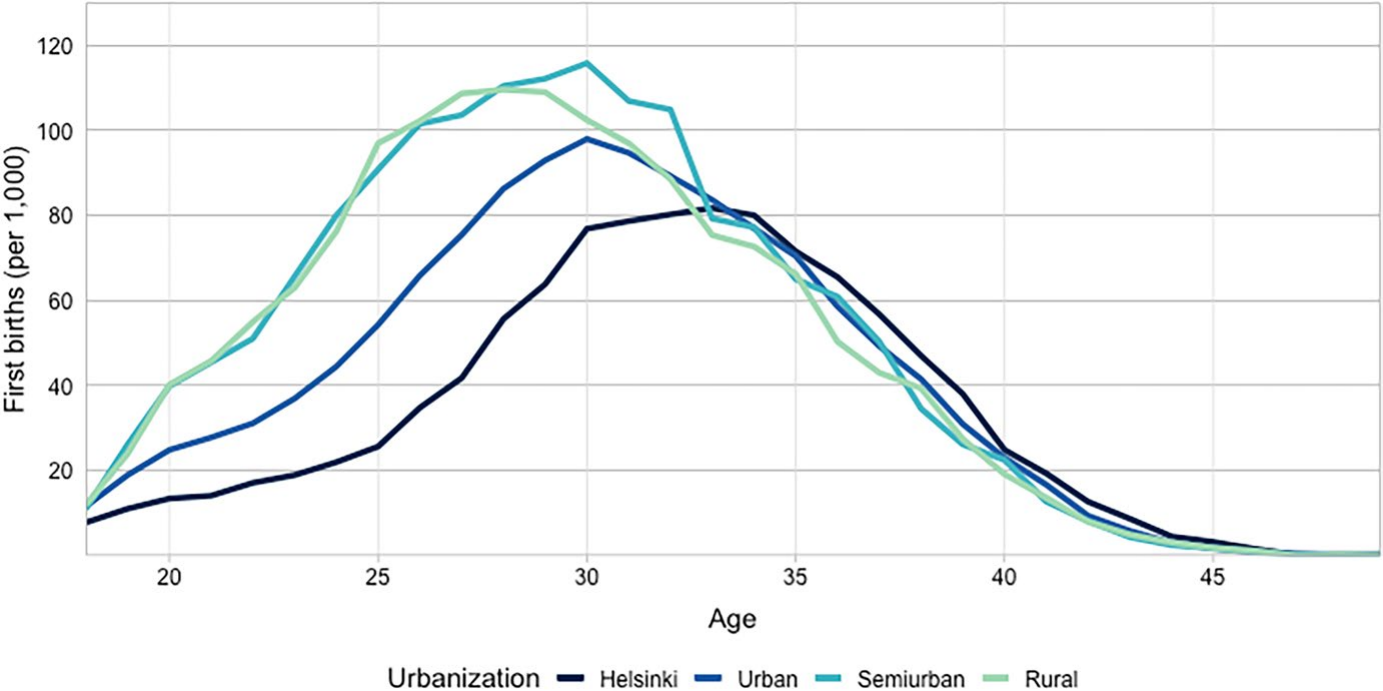
First birth rates (per 1,000 childless women) by municipality level of urbanization, ages 18–49, 2012–2018

First, we examine variation in age-specific first birth rates by level of urbanization and variation in rates by economic activity. This allows us to get a first insight into how the transition to parenthood in Finland varies across these two dimensions over age. First birth schedules in Finland vary substantially between levels of urbanization (Fig. 1). Clearly, first birth rates are highest in rural and semiurban municipalities, especially at younger ages. First birth rates in these areas with rather low levels of urbanization increase quickly and peak at around ages 28 to 30 before they decline sharply. In contrast, first birth rates among women living in Helsinki are very low until after age 25. This is likely related to the fact that during these younger ages many women in Helsinki are enrolled in post-secondary education. After ages 23–25, when we can expect most post-secondary education to have finished, we observe a sharp increase in first birth rates in Helsinki and urban municipalities. Nevertheless, through the ages between 23 and the early 30 s first birth rates in these two most urbanized types of municipalities stay below those for the more rural areas, and Helsinki remains the level of urbanization with the lowest first birth rates. While we see substantial variation in first birth rates by level of urbanization prior to age 33, levels and trends are very similar across the urban–rural gradient after this age.

**Fig. 2:**
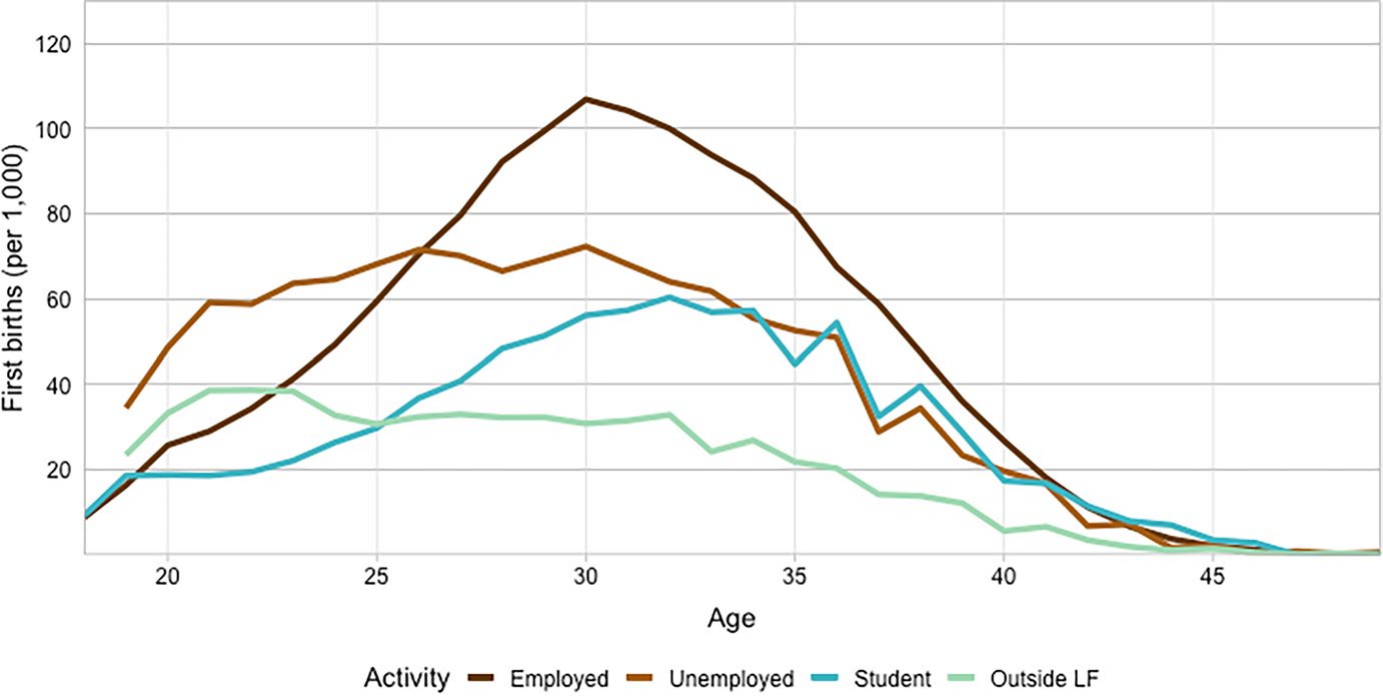
First birth rates (per 1,000 childless women) by economic activity, selected ages 18–49, 2012–2018

Variation in age-specific first birth rates by economic activity is displayed in Fig. 2. At younger ages, first birth rates are the highest among unemployed women and the lowest among students. However, employed women have the highest first birth rates after age 26 and maintain the highest rate throughout the majority of the following ages until after age 41, when first birth rates among all economic activities converge, probably due to small and selective sample sizes (Table 1). The increase in first birth rates among students who are in their later 20 s and early 30 s is primarily related to small sample sizes of students at these ages. Low first birth rates to students at younger ages are likely explained by fertility postponement until finishing education (e.g., Kulu & Washbrook, 2014).

### Multi-Level Analysis of First Birth Hazards

Table 2 shows the results of multi-level discrete-time event history models on the conditional probability of a woman having a first birth in Finland between 2012 and 2018. Results for women of all ages are shown in Models 1–2, for those under age 30 in Models 3–4, and for those ages 30 or older in Models 5–6. Models 1, 3, and 5 only include individual-level controls. The introduction of municipality-level controls (Models 2, 4, and 6) does not greatly change the estimates of the individual-level variables.^1^ Therefore, we focus on the results related to the key variables of interest in the full models (Models 2, 4, and 6).

**Table 2:** Average marginal effects from multi-level discrete-time event history models on the probability of a first birth to women living in Finland by age, 2012–2018. *Source*: Population register of Finland

		Model 1	Model 2	Model 3	Model 4	Model 5	Model 6
		All ages	All ages	Ages 18–29	Ages 18–29	Ages 30–49	Ages 30–49
*Individual-level variables*							
Economic activity	Employed	Ref	Ref	Ref	Ref	Ref	Ref
	Unemployed	0.021 ***	0.017 ***	0.032 ***	0.024 ***	− 0.002 *	− 0.002 *
	Student	− 0.008 ***	− 0.006 ***	− 0.008 ***	− 0.006 ***	− 0.003 **	− 0.003 **
	Not in Labor Force	− 0.012 ***	− 0.009 ***	− 0.004 ***	− 0.003 ***	− 0.026 ***	− 0.024 ***
Household	Less than 20,000	− 0.052 ***	− 0.042 ***	− 0.071 ***	− 0.053 ***	− 0.021 ***	− 0.019 ***
income	20,000 – 38,000	− 0.028 ***	− 0.022 ***	− 0.040 ***	− 0.030 ***	− 0.011 ***	− 0.010 ***
	38,000 – 50,000	Ref	Ref	Ref	Ref	Ref	Ref
	50,000 – 64,000	0.025 ***	0.020 ***	0.034 ***	0.027 ***	0.015 ***	0.013 ***
	64,000 or more	0.060 ***	0.049 ***	0.065 ***	0.050 ***	0.036 ***	0.033 ***
Education	Low	Ref	Ref	Ref	Ref	Ref	Ref
	Medium	− 0.020 ***	− 0.016 ***	− 0.027 ***	− 0.020 ***	− 0.001	− 0.001
	High	− 0.008 ***	− 0.006 ***	− 0.021 ***	− 0.016 ***	0.016 ***	0.015 ***
Country of birth	Native	Ref	Ref	Ref	Ref	Ref	Ref
	Foreign born	0.029 ***	0.023 ***	0.035 ***	0.026 ***	0.022 ***	0.020 ***
Moved in year	Did not move	Ref	Ref	Ref	Ref	Ref	Ref
	Moved	− 0.004 ***	− 0.003 ***	− 0.007 ***	− 0.006 ***	0.004 ***	0.004 ***
Age	18–19	− 0.039 ***	− 0.031 ***	− 0.043 ***	− 0.032 ***		
	20–24	− 0.016 ***	− 0.013 ***	− 0.018 ***	− 0.014 ***		
	25–29	Ref	Ref	Ref	Ref		
	30–34	0.001 ***	0.001 ***			Ref	Ref
	35–39	− 0.033 ***	− 0.026 ***			− 0.029 ***	− 0.027 ***
	40–44	− 0.117 ***	− 0.094 ***			− 0.103 ***	− 0.096 ***
	45–49	− 0.263 ***	− 0.211 ***			− 0.234 ***	− 0.218 ***
*Municipality-level variables*							
Level of urbanization	Helsinki		Ref		Ref		Ref
	Urban		0.012 ***		0.017 ***		0.008 ^#^
	Semiurban		0.017 ***		0.023 ***		0.008 ^#^
	Rural		0.014 ***		0.021 ***		0.007
Unemployment ratio			0.019 *		0.031 **		− 0.040 **
Total fertility rate			0.019 ***		0.020 ***		0.015 ***
Population density (log)			− 0.000		− 0.001 **		0.001 *
Intercept		0.059 ***	0.046 ***	0.062 ***	0.045 ***	0.053 ***	0.049 ***
Random effect’s standard deviation (σ_u_)		0.176	0.083	0.225	0.099	0.142	0.093
Number of person-years		3,414,817	3,414,817	2,221,678	2,221,678	1,193,139	1,193,139
Number of observations		691,687	691,687	515,199	515,199	176,488	176,488
Log Likelihood		− 565,388	− 565,388	− 360,147	− 359,735	− 202,645	− 202,544

Models control for year. ^#^ *p* < 0.10; * *p* < 0.05; ** *p* < 0.01; *** *p* < 0.001

#### First Birth Hazards of Women in Finland

First, we discuss the model results for women of all ages (Model 2, Table 2). Looking at individual-level economic activity, unemployed women have a two percentage point higher annual probability of a first birth (or a 40 percent increased odds in Appendix B Table B.3^2^) of a first birth than employed women. This finding is in line with our expectations, descriptive results, and previous research (Miettinen & Jalovaara, 2020).

However, higher probabilities among unemployed women than their employed counterparts only emerged after accounting for household income.^3^ Within household income, we find a positive gradient; the probability of a first birth is higher in households with higher income. Additionally, women with low levels of education have the highest first birth probabilities, whereas those with medium levels of education are the least likely to have a first child. Regarding the municipality level of urbanization, we find that first birth probabilities are lowest in Helsinki and highest in semiurban and rural areas, which have around 1.7 and 1.4 percentage points higher probability of a first birth than Helsinki, respectively. Finally, the outcomes for municipality-level proportion unemployed show that if unemployment is higher in a municipality, individuals’ first birth probabilities are also higher by about two percentage points for each unit increase in the municipality-level unemployment ratio.

Regarding the control variables, we find the expected inverted u-shaped age-pattern of the transition to first birth, and those who experienced a move in the previous year are less likely to have a first child compared to those who did not move. Foreign-born women are more likely to transition to a first birth compared to natives. Finally, the municipality-level total fertility rate is related to an increased probability of a first birth, while a one unit increase in the municipality-level population density variable is related to almost no change in the probability of a first birth.

#### First Birth Hazards of Women Under Age 30 Versus Women Ages 30 or Older

In contrasting the models for women under age 30 (Model 4, Table 2) and women ages 30 or older (Model 6, Table 2), we will focus on those variables that are most relevant for our main research questions (economic and socio-economic variables, and level of urbanization). We find among women under age 30 (Model 4) similar patterns as we did in the previous step of the analysis (Fig. 2), in which unemployed women displayed higher rates of first births than employed women at younger ages. Young unemployed women in Model 4 exhibit the highest probability of a first birth, with over a two percentage point higher annual probability (or 60 percent increased odds) of a first birth than young employed women. This is also similar to the results for all ages, which is not surprising as most first births in Finland occur below age 30 (Table 1). In the model for women ages 30 or older, these coefficients are negative and not as strong, although still significant (Model 6). The annual probability of a first birth to older unemployed women is less than one percentage point lower than to their employed counterparts. Again, this is in line with Fig. 2 in which we observed more variation in first birth rates at younger ages than at older ages.

A similar change in sign is visible in the variable controlling for municipal-level unemployment. Among younger women, a one unit increase in the municipality-level proportion unemployed is related to a three percentage point higher annual probability (or 110 percent increased odds), while it is related to a four percentage point lower annual probability among older women. For household income we obtain for both younger and older women a positive gradient, in which the probability of a first birth increases with household income.  However, this gradient is more pronounced among younger compared to older women. As for education, we again see strong differences between the model for younger women and the model for older women. While among younger women the low educated have the highest probability to have a first birth, it is the highly educated who are more likely to do so among older women. It is also worth noting that women ages 30 or older who moved in the last year had a relatively high probability of a first birth, although this is negative for women under age 30.

The outcomes for the municipality level of urbanization variable are in line with the first birth rates depicted in Fig. 1. In the model for younger women, we see substantial differences between Helsinki and the other three categories, with Helsinki displaying the lowest first birth probabilities. Among women under age thirty, the probability of a first birth was highest for those living in semiurban municipalities. Among older women, we still obtain the lowest probability for Helsinki, but the differences among the levels of urbanization are smaller.

### The Probability of a First Birth by Economic Activity and Level of Urbanization

**Fig. 3:**
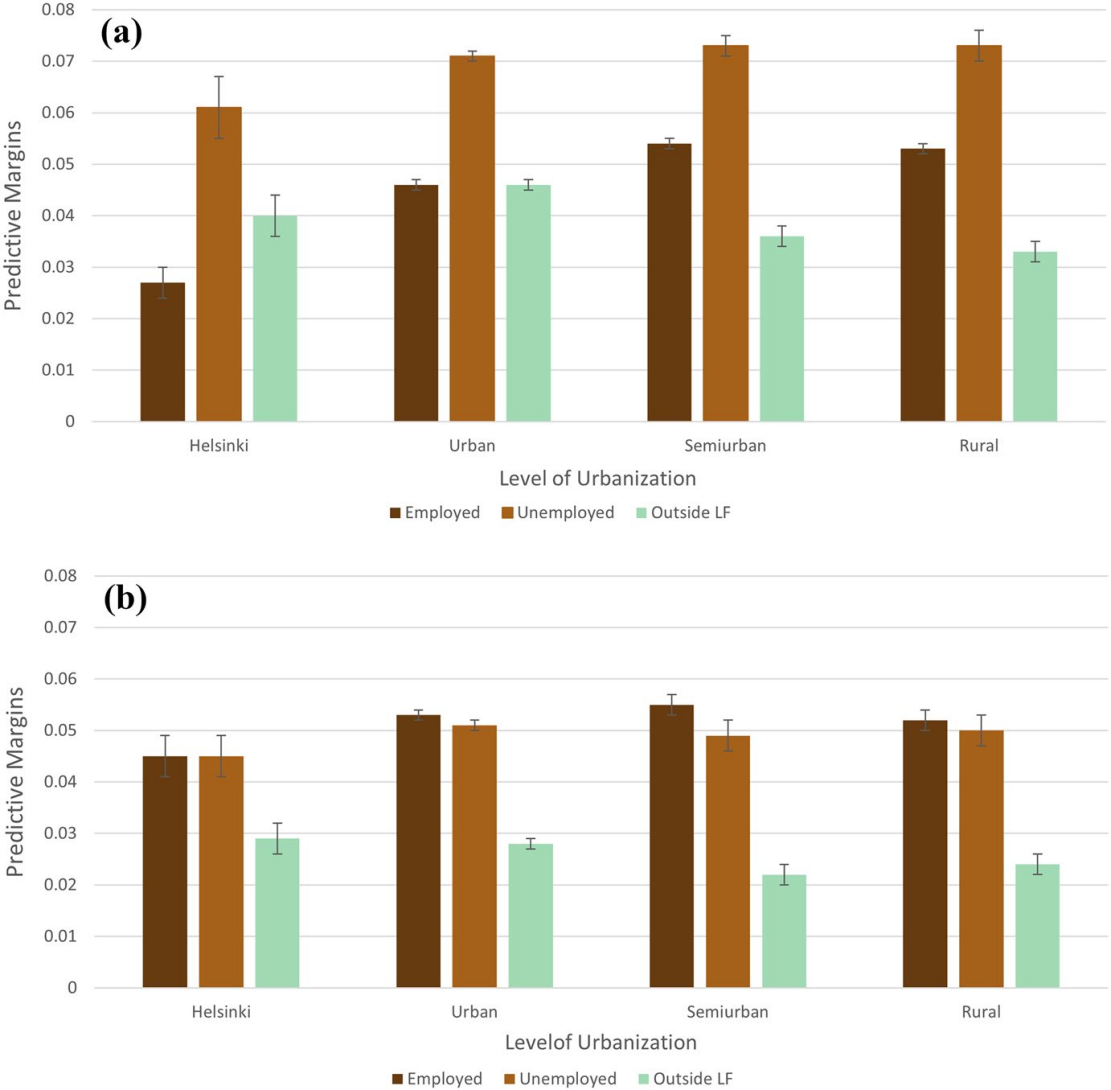
Predictive margins of economic activity, not showing students, on the probability of having a first birth by level of urbanization for **a** women aged 18–29 and **b** women ages 30–49, 2012–2018. Notes: Appendix B Table B.1 shows results from these models displayed as average marginal effects. Models control for age, education, country of birth, recent move, household income, municipality proportion unemployed, municipality TFR, municipality population density, year, and random effects of municipality

Figure 3 shows the predictive margins of the interaction between economic activity and level of urbanization.^4^ Figure 3a displays the results for women aged 18 to 29 and Fig. 3b for women ages 30 to 49.^5^

Young, employed women show the strongest urban–rural gradient in first birth probability: the first birth probability among employed women increases as we move from Helsinki to urban and semiurban/rural municipalities (Fig. 3a). We observe a weaker urban–rural gradient among young, unemployed women: Helsinki displays the lowest first birth probability, but there are small to no differences between urban, semiurban, and rural municipalities. It is worth noting, however, that, across all levels of urbanization, those women under age 30 who are unemployed have by far the highest first probabilities. Among young women who are outside of the labor force the urban–rural gradient is rather opposite with semiurban and rural municipalities showing lower first birth probabilities than urban areas and Helsinki. This results in a crossover among employed women and women outside of the labor force. While first birth probabilities in semiurban and rural levels of urbanization are higher among young, employed women than among young women outside the labor force, in urban areas young, employed women and those outside the labor force have equal first birth probabilities and in Helsinki those who are outside the labor force are more likely to have a first birth than those who are employed.

Among women ages 30 or older, we generally find smaller differences in first birth probabilities across the four urbanization levels and little evidence of urban–rural gradients (Fig. 3b). Employed and unemployed women ages 30 or older are similarly likely to have a first child across the levels of urbanization, whereas those outside the labor force are less likely to do so across all levels of urbanization. Compared to younger women, the first birth probabilities for women ages 30 or older are smaller for those who are unemployed or outside of the labor force. This is true across all levels of urbanization. The differences between the two age groups for employed women, however, are more varied. First birth probabilities among older women are greater than those for younger women in Helsinki and urban municipalities. In Helsinki they are almost double. In semiurban and rural areas, on the other hand, older and younger employed women do not differ strongly in first birth probabilities. Women ages 30 or older and outside of the labor force display a probability of a first birth much lower than the other two economic activities across all levels of urbanization, unlike the trend among similar young women noted previously.

## Discussion and Conclusion

In this paper, we took a detailed look at the urban–rural dimension of recent fertility variation in Finland —an aspect which has so far received little attention in research on the Nordic fertility decline. We then explored how employment status, one dimension of economic uncertainty theories, varies in its relationship with fertility across levels of urbanization to better understand how these two aspects of fertility conditions are related. We focused on economic circumstances and related uncertainty as these aspects have been identified as important drivers of fertility decline in the existing literature.

Combining detailed individual-level Finnish register data with aggregate-level data at the municipality level allowed us to account for both individual-level and contextual-level aspects and conditions. Looking at these relationships by age allowed us to explore differences between women under age 30 and women ages 30 or older.

As we expected, the level of urbanization is highly relevant for variation in first birth probabilities in Finland. However, our results clearly show that most of the urban–rural variation is concentrated at younger ages, since we did not observe strong differences across levels of urbanization for women ages 30 or older. This is in line with previous aggregate-level findings in Finland (Campisi et al., 2022). While women of younger ages can be negatively affected by unemployment in their fertility decisions (Goldstein et al., 2013), the differences across levels of urbanization are certainly not only driven by uncertainty. Higher education institutions, for example, are more concentrated in urban areas. As a result, postponement at younger ages due to attending higher education is more likely to depress first birth probabilities in highly urbanized areas (also due to selective migration) than in more rural areas. However, we did not find strong evident of recuperation in these levels of urbanization among results for women ages 30 or older, possibly related to out-migration from urban areas with the intentions of childbearing (the ‘family phase’ in Dommermuth & Klüsener, 2019).

However, for women under age 30 we found a rather strong positive urban–rural gradient among employed women and a slight positive urban–rural gradient among unemployed women, in line with economic considerations. An issue in interpreting this pattern is that this gradient is not necessarily driven by uncertainty but might also be the result of variation in living costs. While the addition of household income, which may help mitigate living costs, increased the probabilities of a first birth among all economic activities and age groups, the fact that we do not see positive urban–rural gradients among employed and unemployed women ages 30 or older suggests that it is not living costs alone which are driving the urban–rural gradients in first birth probabilities. That uncertainty plays a role is also underlined by a study by Savelieva et al. (2022) based on Finnish survey data. The authors show that postponement of births due to uncertainty is particularly likely to occur in the Helsinki metropolitan area, with the occurrence decreasing over age. Additionally, for young people, it tends to be more difficult to establish themselves in big cities, which might suppress fertility at younger ages (Kulu & Washbrook, 2014). Here, it would be interesting to contrast our outcomes with model results for periods prior to the Nordic fertility decline to see whether the urban–rural gradients among young women existed previously as well. This would also be an interesting avenue for future research.

The positive urban–rural gradients we observe among employed and unemployed women below age 30 are most applicable to those living in Helsinki and, to a lesser extent, urban municipalities, while semiurban and rural displayed generally similar first birth probabilities. In this way, Helsinki emerged as a unique municipality context, even compared to other urban municipalities. We believe that the rather high first birth probabilities in semiurban municipalities compared to more urban areas are related to the characteristics of suburbs which are conducive for fertility (Kulu, 2013; Kulu & Vikat, 2007).

As register data do not contain information on perceptions of uncertainty, we had to measure this dimension indirectly via proxy variables such as employment status, income, and municipal-level proportion of unemployed. Our finding that unemployed women showed higher first birth probabilities at younger ages compared to employed women and those outside of the labor force might at first sight be considered counterintuitive to the uncertainty argument. However, this is in line with our expectations and previous findings for Finland (Miettinen & Jalovaara, 2020; Vikat, 2004) and other contexts (Kreyenfeld & Andersson, 2014; Özcan et al., 2010). It has been suggested that at younger ages, unemployed women may have fewer barriers to fertility such that short-term unemployment may, in this group, not be seen as a disruption to long-term life plans, and thus fertility desires may not be viewed as competing with other career or education ambitions. Also supporting the barriers argument, we observe swift changes in first birth rates around ages when we would expect specific life course transitions. For example, large increases in first birth rates to many women occurred around age 19, after leaving school, and age 23, after leaving university. It seems that when barriers to fertility arise, such as career paths or additional schooling, fertility probabilities decrease, and fertility may be further postponed. For example, employed women did not display the highest first birth rate until after age 26. Young, employed women may not desire to have children if they believe parenthood will take away opportunities at the beginning of their careers. This might contribute to our result that young, employed women displayed lower first birth probabilities than unemployed women. Beyond this mechanism, it has been suggested that the strong social welfare system in Finland may also serve to reduce the impact of lost earnings for unemployed women or families through unemployment benefits (Miettinen & Jalovaara, 2020). Similar mechanisms might also explain why we found a positive relationship between the municipal-level proportion unemployed and first birth risks among younger women.

The outcomes for income both for younger and older women are in line with uncertainty considerations. The first birth risk is increasing with rising income, and this increase is particularly steep among younger women. This fits with findings by others that the fertility of young women is particularly affected by uncertainty (e.g., Goldstein et al., 2013). Furthermore, additional revenues of income (e.g., through the partner) can also reduce the negative impact of individual unemployment on first birth probabilities by reducing the financial burden of unemployment. In this way, unemployment may not impact fertility as much if the woman’s partner still receives income (Busetta et al., 2019). Sensitivity checks suggest that mechanisms exist in this direction: the addition of household income increased the probability of a first birth to an unemployed woman in both age groups. Again, this might be related to the economic situation of the partner. Unfortunately, the scope of this paper did not allow us to look in detail at the partner. But it seems to be a fruitful avenue for future research to perform similar analyses that also include detailed information on the partner.

There is also a dichotomy between labor force participation, either as employed or unemployed, and otherwise not being in the labor force. Labor force participation of any sort (employed, unemployed) is related to higher first birth probabilities across almost all ages and levels of urbanization than not participating in the labor force. The only exceptions are young women in Helsinki and urban municipalities, where employed women have a, respectively, similar or lower first birth probability than those outside of the labor force. These mixed findings for persons outside of the labor force require further investigation. One potential explanation might be the unobserved heterogeneity of those outside of the labor force. For instance, those who are outside of the labor force for medical reasons may also experience infertility for the same reasons that place them outside of the labor force. We also do not control for specific or varying migration backgrounds, such as in the prevalence of traditional gender patterns where the women tend to stay out of the labor market to focus on family-related activities. Future research should investigate such potential differences between women in and outside of the labor force in more detail. Another limitation of the register data we used is that we could not fully capture the complexity of fertility decisions. Another dimension that we were only able to cover to a limited degree is the timing of fertility in relation to other life course events. It would be fruitful to compare our results to those using monthly data, which can better capture transitions between economic activities or levels of urbanization (e.g., migration). Capturing internal migration with fertility timing simultaneously would provide greater insight into who is having children and the characteristics of these individuals that may or may not be changing over time.

Nonetheless, we demonstrated that the addition of the so-far underexplored urban–rural dimension in research on the Nordic fertility decline leads to new important insights on the possible determinants of this process. Our results suggest that young women living in big cities and urban municipalities should particularly be in the focus of research aimed at understanding fertility trends in Finland and the role of uncertainty in fertility variation.

## Data Availability

Not applicable.

## Appendix

### Additional Information on Variables

Level of urbanization classification is developed by Statistics Finland, which differentiates three types of regions: urban, semiurban, and rural areas. We consider Helsinki separately from urban municipalities due to its unique nature as the most densely populated city and the capital city. Urban municipalities are those in which at least 90 percent of the population lives in urban settlements, or the largest settlement has a population of 15,000 or greater. Semiurban municipalities are those in which 60 to 90 percent of the population lives in urban settlements and the largest settlement has a population of 4,000 to 14,999. If the largest settlement has instead a population less than 4,000, such municipalities are classified as rural. In addition, all municipalities in which less than 60 percent of the population lives in an urban settlement and the largest urban settlement has a population of 15,000 or less are also classified as rural. Commuting municipalities for Helsinki are not included in the “Helsinki” category because these municipalities include other cities and their total fertility rates are more similar to those of other “Urban” municipalities than Helsinki.

Age is measured as an individual’s age in years at the beginning of the year.

Educational level is measured as the highest level of educational qualification. Following the ISCED 2011 classification, it is categorized as low (less than upper secondary level (ISCED 1 and 2)), medium (upper secondary level or post-secondary non-tertiary education (ISCED 2 and 4)), or high (short-cycle tertiary education, Bachelor’s or equivalent level, Master’s or equivalent level, Doctoral or equivalent level (ISCED 5 or higher)).

We separate individuals who are foreign born and those born in Finland. These individuals may have been in Finland already at the start of the observation period or migrated to Finland during the observation period.

Individuals are movers each year if their municipality of residence is different from that in the previous year. Movers may have migrated into Finland during the period (prior municipality outside of the country) or migrated within Finland during the period (internal migrants). This variable cannot capture moves that occurred within the same municipality and whether a move occurred before or after a birth during the same year. However, capturing the timing of a move relative to birth is not expected to have a large impact on the results, as those who move due to childbearing may move in anticipation of or in response to a birth (Dommermuth & Klüsener, 2019; Kulu & Washbrook, 2014).

Municipality-level variables are calculated for each municipality and each year using information on the last day of the previous year on all individuals within Finland. There are 310 municipalities in Finland. Some issues of endogeneity may arise from calculating municipality-level variables using individual-level data (Kravdal, 2003). However, these issues largely stem from the application of individual-level errors to the municipality level. We do not expect this to be a large issue since municipality-level variables were calculated using a larger sample than the analytical sample. The sample sizes for calculating each variable are noted below.

Fertility levels are measured annually using the total fertility rate. It is calculated as the sum of age-specific fertility rates using five-year age groups for women aged 15 to 49, multiplied by five. 1,391,130 women between the ages 15 and 39 were used to calculate the municipality total fertility rate.

Population density is measured as the municipality population per square kilometer, and it is calculated by dividing the total population living in the municipality at the end of the year by square kilometer of municipality area. Municipality areas in 2017 were used to calculate population density for all years. Municipality areas did not change much from year to year. 5,898,518 males and females of all ages were used to calculate municipality population density. The log of population density is used in the analyses to reduce the effect of outliers (e.g., very large metropolitan areas such as Helsinki).

### Discrete-Time Event History Analysis of the Hazard of a First Birth

Discrete-time event history analysis treats the occurrence of an event as a binary outcome, where 1 is the occurrence of an event and 0 is no occurrence. To do so, discrete-time event history models can be estimated using logistic regression for the conditional probability that the event occurs during the period, given that it has not occurred before. The probability of an event then ranges between zero and one. The equation for an event history model with log probability is outlined below:

$$\log \left[ {\frac{{p_{ijt} }}{{1 - p_{ijt} }}} \right] = \alpha_{t} + \beta X_{ijt}$$

where the odds of a first birth (*p*_*it*_) for individual *i* in municipality *j* during year *t* is expressed as the log of the probability divided by the probability complement (*1- p*_*it*_). The log odds are estimated by the individual-level variables *X*_*i*_.

However, discrete-time event history models do not inherently account for the clustering of individuals across space. The clustering of individuals across space, here municipalities, can bias regression estimates through spatial autocorrelation of the residual errors (Baltagi & Li, 2004). To overcome the issue of spatial clustering, it is appropriate to use discrete-space multilevel models. A multilevel analysis is considered appropriate for this analysis because the number of municipality groups is large (310 municipalities) (Bryan and Jenkins, 2015). In multilevel modeling, individuals are nested within municipalities. Nesting is achieved through the inclusion of a regression term for municipality of residence. A random term for the effect of municipalities is included.

A random effects model is applied, rather than a fixed effects model, because the random effects model allows us to explore the role of space in fertility. Using a fixed effects model would remove municipality-level differences from estimation. For example, fixed effects results of level of urbanization would reflect the role of changing level of urbanization over time, while random effects of urbanization reflect the role of differing levels of urbanization between municipalities as well as changes in urbanization over time. While significant results from Hausman (1978) tests suggest that orthogonal assumptions of random effects may be violated in our sample and that fixed effects models are preferred, we continue with random effects models due to theoretical motivations outlined above.

Both individual-level and municipality-level characteristics are included to estimate the probability of a live birth. The equation for the multilevel effects discrete-time event history model is outlined below:

$$\log \left[ {\frac{{p_{ijt} }}{{1 - p_{ijt} }}} \right] = \alpha_{t} + \beta X_{ijt} + \beta M_{jt} + year + \phi_{j}$$

where *p*_*i*_ is the probability of the outcome of a first birth for individual *i* in municipality *j* is again expressed as a log. The log odds of a first birth are estimated by the individual-level variables *X*_*ijt*_ and municipality-level variables *M*_*jt*_. Fixed effects for the year are included to account for systematic period effects in changing fertility over time. *ϕ*_*j*_ is the random effect of municipality. The aim of the multilevel event history models is to understand how the log odds of a first birth vary by both individual-level and municipality-level characteristics.

Multilevel event history models are calculated first for women of all ages 18 to 49. Then, separate models are conducted for women who are aged 18 to 29 and for women who are ages 30 to 49. Proportionality assumptions on the distribution of first births are not met and so two separate analyses are conducted by age group.

The results of the models are shown as average marginal effects. Average marginal effects provide the best comparability between results of differing models and samples (Mood, 2010). Average marginal effects for each variable are calculated as the average effect of each person-year observation in the sample. For example, an average marginal effect of 0.10 reflects a ten percent average change in the annual probability of a first birth.

### Appendix B–Further Figures and Tables

See Tables 3,

**Table 3:** Average marginal effects from interaction models, 2012–2018. *Source*: Population register of Finland

		Ages 18–29	Ages 30–49
Economic activity *	Employed * Helsinki	Ref	Ref
Municipality-level urbanization	Employed * Urban	0.019 ***	0.008 *
	Employed * Semiurban	0.027 ***	0.010 *
	Employed * Rural	0.025 ***	0.007
	Student * Helsinki	0.002 *	- 0.002
	Student * Urban	0.013 ***	0.005
	Student * Semiurban	0.015 ***	0.006
	Student * Rural	0.009 **	0.008
	Unemployed * Helsinki	0.033 ***	0.003
	Unemployed * Urban	0.044 ***	0.007
	Unemployed * Semiurban	0.046 ***	0.004
	Unemployed * Rural	0.046 ***	0.005
	Outside LF * Helsinki	0.012 ***	- 0.016 ***
	Outside LF * Urban	0.018 ***	- 0.017 ***
	Outside LF * Semiurban	0.009 *	- 0.023 ***
	Outside LF * Rural	0.005	- 0.021 ***
Household income	Less than 20,000	- 0.053 ***	- 0.019 ***
	20,000 – 38,000	- 0.030 ***	- 0.010 ***
	38,000 – 50,000	Ref	Ref
	50,000 – 64,000	0.027 ***	0.013 ***
	64,000 or more	0.051 ***	0.034 ***
Education	Low	Ref	Ref
	Medium	- 0.020 ***	- 0.001
	High	- 0.016 ***	0.014 ***
Country of birth	Native	Ref	Ref
	Foreign born	0.025 ***	0.020 ***
Moved in year	Did not move	Ref	Ref
	Moved	- 0.005 ***	0.003 ***
Age	18–19	- 0.032 ***	
	20–24	- 0.014 ***	
	25–29	Ref	
	30–34		Ref
	35–39		- 0.027 ***
	40–44		- 0.096 ***
	45–49		- 0.218 ***
Municipality unemployment ratio		0.038 ***	- 0.039 **
Municipality total fertility rate		0.020 ***	0.015 ***
Municipality population density (log)		- 0.001 **	0.001 *
Intercept		0.045 ***	0.049 ***
Random effect’s standard deviation (σ_u_)		0.098	0.093
Number of person-years		2,221,678	1,193,139
Number of observations		515,199	176,488
Log Likelihood		- 359,503	- 202,523

Models control for year. ^#^ *p* < 0.10; * *p* < 0.05; ** *p* < 0.01; *** *p* < 0.001

**Table 4:** Average marginal effects of the probability of a first birth to women living in Finland by age, 2012–2018. *Source*: Population register of Finland

		All ages	Ages 18–29	Ages 30–49
*Individual-level variables*				
Economic activity	Employed	Ref	Ref	Ref
	Unemployed	- 0.002 **	0.011 ***	- 0.018 ***
	Student	- 0.028 ***	- 0.030 ***	- 0.021 ***
	Not in Labor Force	- 0.033 ***	- 0.023 ***	- 0.039 ***
Age	18–19	- 0.077 ***	- 0.086 ***	
	20–24	- 0.038 ***	- 0.042 ***	
	25–29	Ref	Ref	
	30–34	0.011 ***		Ref
	35–39	- 0.018 ***		- 0.025 ***
	40–44	- 0.099 ***		- 0.093 ***
	45–49	- 0.242 ***		- 0.217 ***
Intercept		0.056 ***	0.061 ***	0.049 ***
Random effect’s standard deviation (σ_u_)		0.009	0.216	0.010
Number of person-years		691,687	515,199	176,488
Number of observations		3,414,817	2,221,678	1,193,139
Log Likelihood		- 582,230	- 372,759	- 206,689

Models control for year. ^#^ *p* < 0.10; * *p* < 0.05; ** *p* < 0.01; *** *p* < 0.001

^4^,

**Table 5:** Odds Ratios of the probability of a first birth to women living in Finland, 2012–2018. *Source*: Population register of Finland

		All ages	Ages 18–29	Ages 30–49
*Individual-level variables*				
Economic activity	Employed	1	1	1
	Unemployed	1.406 ***	1.626 ***	0.938 **
	Student	0.856 ***	0.862 ***	0.964 ^#^
	Not in Labor Force	0.785 ***	0.930 ***	0.502 ***
Household income	Less than 20,000	0.270 ***	0.224 ***	0.536 ***
	20,000 – 38,000	0.597 ***	0.548 ***	0.756 ***
	38,000 – 50,000	1	1	1
	50,000 – 64,000	1.389 ***	1.433 ***	1.349 ***
	64,000 or more	1.981 ***	1.850 ***	1.899 ***
Education	Low	1	1	1
	Medium	0.684 ***	0.616 ***	0.981
	High	0.866 ***	0.686 ***	1.389 ***
Country of birth	Native	1	1	1
	Foreign born	1.720 ***	1.886 ***	1.572 ***
Moved in year	Did not move	1	1	1
	Moved	0.930 ***	0.874 ***	1.088 ***
Age	18–19	0.478 ***	0.455 ***	
	20–24	0.745 ***	0.718 ***	
	25–29	1	1	
	30–34	1.026 ***		1
	35–39	0.537 ***		0.544 ***
	40–44	0.109 ***		0.116 ***
	45–49	0.007 ***		0.007 ***
*Municipality-level variables*				
Level of urbanization	Helsinki	1	1	1
	Urban	1.376 ***	1.638 ***	1.201 ^#^
	Semiurban	1.509 ***	1.848 ***	1.217 ^#^
	Rural	1.442 ***	1.769 ***	1.167
Unemployment ratio		1.551 *	2.135 **	0.407 **
Total fertility rate		1.581 ***	1.634 ***	1.408 ***
Population density (log)		0.993	0.976 **	1.022 *
Intercept		0.041 ***	0.039 ***	0.041 ***
Random effect’s standard deviation (σ_u_)		0.083	0.099	0.093
Number of person-years		3,414,817	2,221,678	1,193,139
Number of observations		691,687	515,199	176,488
Log Likelihood		- 565,388	- 359,735	- 202,544

Models control for year. ^#^ *p* < 0.10; * *p* < 0.05; ** *p* < 0.01; *** *p* < 0.001

^5^.

## Footnotes

1 Consistency between the individual and multilevel models is likely related to the municipality-level random effects included in Models 1, 3, and 5.

2 Appendix B Table B.3 shows the odds ratios of the probability of a first birth to women living in Finland, 2012–2018.

3 See Appendix B Table B.2 for regression models without income.

4 Likelihood ratio tests indicate that interaction models in Fig. 3a (χ^2^(9) = 282.8, p < 0.000) and Fig. 3b (χ^2^(9) = 337.7, p < 0.000) are significant.

5 See Appendix B Table B.1 for full results of the interaction models displayed as average marginal effects, not predictive margins, for transparency.
